# Removing scanner effects with a multivariate latent approach: A RELIEF for the ABCD imaging data?

**DOI:** 10.1162/imag_a_00157

**Published:** 2024-05-02

**Authors:** Dominik Kraft, Gloria Matte Bon, Édith Breton, Philipp Seidel, Tobias Kaufmann

**Affiliations:** Department of Psychiatry and Psychotherapy, Tübingen Center for Mental Health, University of Tübingen, Tübingen, Germany; Department of Women’s and Children’s Health, Science for Life Laboratory, Uppsala University, Uppsala, Sweden; Centre for Precision Psychiatry, Division of Mental Health and Addiction, Institute of Clinical Medicine, University of Oslo, Oslo, Norway; German Center for Mental Health (DZPG), partner site Tübingen, Tübingen, Germany

**Keywords:** scan site harmonization, ABCD study, RELIEF, ComBat, CovBat, site effects

## Abstract

Scan site harmonization is a crucial part of any neuroimaging analysis when data have been pooled across different study sites. Zhang and colleagues recently introduced the multivariate harmonization method RELIEF (REmoval of Latent Inter-scanner Effects through Factorization), aiming to remove explicit and latent scan site effects. Their initial validation in an adult sample showed superior performance compared to established methods. We here sought to investigate utility of RELIEF in harmonizing data from the Adolescent Brain and Cognitive Development (ABCD) study, a widely used resource for developmental brain imaging. We benchmarked RELIEF against unharmonized, ComBat, and CovBat harmonized data and investigated the impact of manufacturer type, sample size, and a narrow sample age range on harmonization performance. We found that in cases where sites with sufficiently large samples were harmonized, RELIEF outperformed other techniques, yet in cases where sites with very small samples were included there was substantial performance variation unique to RELIEF. Our results therefore highlight the need for careful quality control when harmonizing data sets with imbalanced samples like the ABCD cohort. Our comment alongside shared scripts may provide guidance for other scholars wanting to integrate best practices in their ABCD related work.

Pooling data from different study sites is a common practice in the field of neuroimaging and allows the community to perform large-scale multi-center studies, like the Adolescent Brain and Cognitive Development (ABCD;Casey et al., 2018) study. While combining data from different magnetic resonance imaging (MRI) scanners may increase statistical power, it may also introduce biases due to inter-scanner differences. Such unwanted variation can harm the signal-to-noise-ratio substantially. Several studies have therefore targeted the development of statistical harmonization techniques that attempt to capture and remove inter-scanner confounds (e.g.,Beer et al., 2020;Chen et al., 2022;Fortin et al., 2016,^2018^), yet the task of modeling scanner-related differences while retaining site-specific differences in the signal of interest or in certain covariates is complex and remains challenging.

Recently,Zhang et al. (2023)introduced a novel multivariate data harmonization method RELIEF (REmoval of Latent Inter-scanner Effects through Factorization). This method is based on a simultaneous dimension reduction and interlinked matrix factorization to remove explicit and latent site effects (seeZhang et al., 2023for a detailed description). The authors compared their newly proposed method to three widely used harmonization tools, that is, (1) adjusted residuals, (2) ComBat (Fortin et al., 2018), and (3) CovBat (Chen et al., 2022). In addition to simulations, they tested harmonization performance in a sample of N = 431 participants, including healthy controls and patients with schizophrenia (age = 18–55 years). Imaging data included fractional anisotropy (FA) and mean diffusivity (MD) features from three scanning sites with three different scanners built by two manufacturers (i.e., General Electric (GE) and Siemens). Across different comparisons throughout the paper, RELIEF showed superior performance in mitigating scanner effects and preserving biologically meaningful variation compared to the other methods, suggesting its benefit for harmonization in multi-site neuroimaging studies.

Here, we sought to investigate RELIEF’s performance in the ABCD study. Because this sample is widely used in the neuroimaging community, we here share the gained insights. The ABCD sample has characteristics that differ substantially from the initial RELIEF validation sample. First, it contains a much larger number of subjects, which allows for investigations into sample size differences in data harmonization. Second, it contains much more scan sites that each contributed data from a harmonized protocol, allowing to investigate effects of scanner hardware. Finally, the ABCD comprises much younger individuals, which typically relates to lower image quality due to motion confounds, and a narrow age range per scan timepoint, which allows to investigate how covariates with narrow distributions are modeled during harmonization (Casey et al., 2018).

We benchmarked RELIEF’s (version 0.1.0) performance against unharmonized (raw), ComBat (*neuroCombat*, version 1.0.13), and CovBat (version 0.1.0) harmonized data in R (version 4.2.3). For comparison to the initial RELIEF study (Zhang et al., 2023), we focused on diffusion tensor imaging and utilized 37 FA and MD imaging features as preprocessed by the ABCD study team from the cross-sectional baseline study visit data. In a first analysis which targeted a controlled setting to study harmonization, we limited the included MRI scanners to those manufactured by either Siemens (i.e., Prisma, Prisma Fit) or GE (i.e., Discovery MR750), as those were used by the sites contributing most data in ABCD and to match the comparisons provided inZhang et al. (2023). To strictly control for potential confounding effects stemming from different and unbalanced sample sizes per site, we included 8 study sites (i.e., 8 different MRI devices) for which at least 500 subjects were available and randomly subsampled N = 500 subjects to equalize sample sizes across sites for unbiased comparison (Supplementary Table S1afor demographics). Lastly, we performed ComBat, CovBat, and RELIEF harmonization with MRI device serial number (i.e., site) as batch variable, and age and sex as covariates to be preserved.

As a performance test for data harmonization in this sample size controlled setting, we deployed a classification task, in which we sought to classify MRI site from either raw, ComBat, CovBat, or RELIEF harmonized imaging data (cf.Chen et al., 2022;Zhang et al., 2023). We performed a Quadratic Discrimination Analyses (QDA; default settings in scikit-learn, version 1.3.2.,Pedregosa et al., 2018) with a stratified 10-fold cross-validation in a One-vs-One (i.e., site vs. site) scheme in python (version 3.9.16). For each pair, we defined each scanner as the positive class once and averaged the two area under the curve (AUC) scores to obtain one mean performance per pair. In the classification framework, a more effective harmonization would lead to lower AUC scores closer to the chance level of 50% in the site-versus-site comparison, while unharmonized data or an inefficient removal of scanner biases would still allow distinguishing scanning sites and thus result in higher AUC scores. As expected, compared to unharmonized raw data, all harmonization techniques significantly hamper the model’s ability to classify scan site and thus result in lower AUC scores.

In accordance withZhang et al. (2023), we found that RELIEF-based ML models yielded the lowest AUC scores (i.e., best harmonization performance) both for FA and MD features (Fig. 1, Panel A, C), however with less pronounced effect boosts compared to ComBat and CovBat than what was initially reported byZhang et al. (2023). While harmonization worked well overall, our results illustrated that across brand (blue) comparisons generally yield higher AUC than within manufacturer (green) comparisons. Thus, while effective at removing within-scanner brand confounds, none of the harmonization tools managed to properly mitigate confounds across brands to the same degree, clearly highlighting the need for further development.

**Fig. 1.**
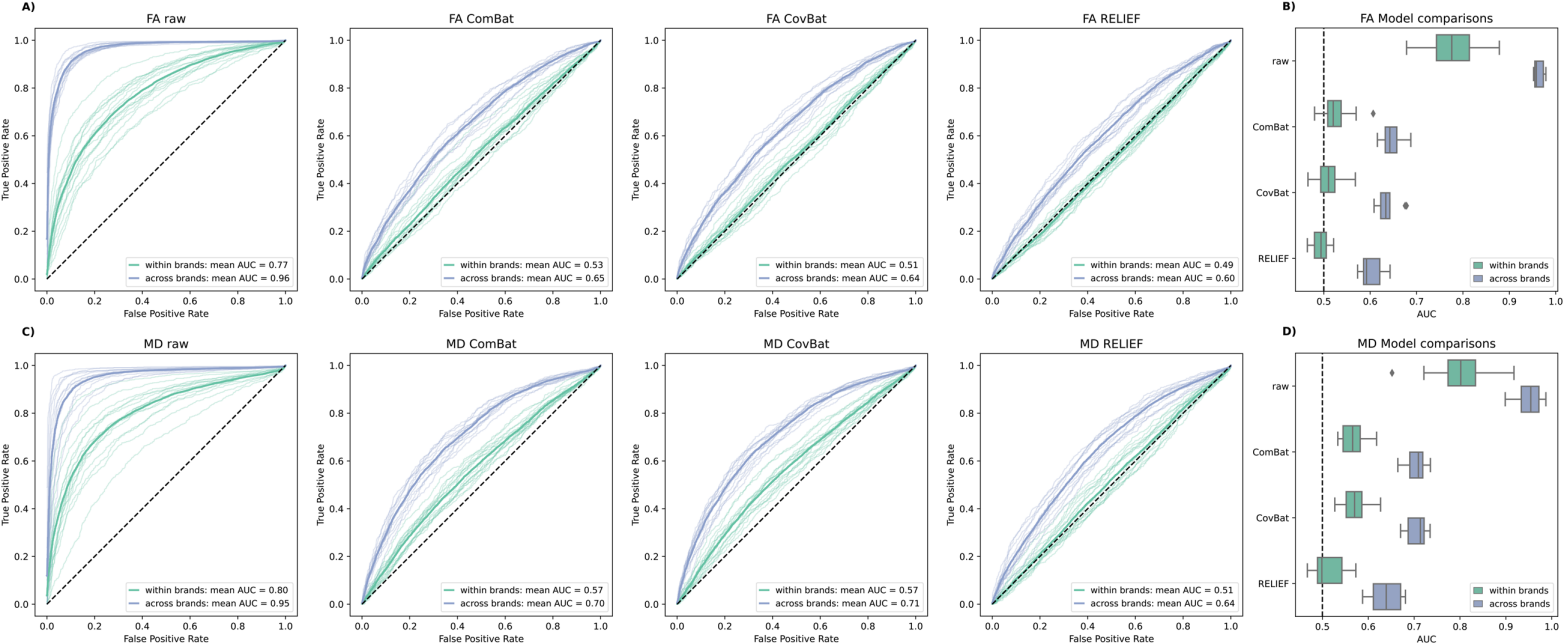
Scan site classification performance across unharmonized and harmonized fractional anisotropy (FA; A) and mean diffusivity (MD; C) data in a controlled subsample of the ABCD with 500 subjects per site. Opaque curves depict pair-wise comparisons (e.g., scanner A1 vs. scanner A2) and are overlaid with thick mean curves for within (green) versus across (blue) manufacturer brand comparison. Mean lines across raw, ComBat, and RELIEF data are combined to allow for a direct comparison (B, D). Model comparison across harmonization techniques. Boxes depict median AUC (vertical line) and interquartile ranges. Note that performance closer to AUC = .5 (chance level) describes a more efficient scan site harmonization and is thus desirable. AUC: area under the curve.

Beyond being capable of effectively removing inter-scanner biases, harmonization methods should also preserve biologically meaningful variance. Thus, to test for the harmonization technique’s ability to preserve biological variance from the covariates (i.e., age and sex), we deployed a simple multivariate machine learning (ML) model aiming at predicting or classifying the covariates age and sex respectively. For age, we performed a Support Vector Regression (SVR) with a 5-fold cross-validation and recorded the correlation between the predicted age and true age as performance metric. For sex, we used a Support Vector Classifier (SVC) together with a stratified 5-fold cross-validation to account for potential class imbalance and obtained balanced accuracy scores to assess the model’s performance. Both SVR and SVC were performed on either raw, ComBat, CovBat, or RELIEF harmonized FA or MD features with default settings in skicit-learn (version 1.3.2.,Pedregosa et al., 2018). Of note, the focus is not the overall performance, but the comparison between unharmonized and harmonized model performance. We hypothesized that if the harmonization techniques can preserve biological variance related to the covariates, the resulting ML models should perform equally well as models based on raw (i.e., unharmonized) data. We performed the machine-learning analyses in two settings—single versus mixture sites—to test whether ML performance and variance changes as a function of sample decomposition. Specifically, for the single site setting, we subsampled n = 496 participants from each site and deployed either the SVR or SVC model depending on the target variable and report mean and standard deviations across all 8 sites for the respective performance metric. In the mixed-sites setting, we subsampled n = 62 subjects from each of the 8 sites, yielding again a sample of n = 496 participants (i.e., 8 x 62). The rationale for the number n = 496 is that it is closest to the initial analysis of n = 500, while allowing us to mix equally sized subsets from 8 sites. Mixed-sites performance metrics were averaged across 500 random seeds and reported alongside their standard deviation. We hypothesized that performance should not drop for the mixture versus single site setting after effective harmonization of site effects. Indeed, in both single and mixed site settings, models based on harmonized data yielded numerically very similar performance metrics compared to raw features (seeTable 1), indicating that ComBat, CovBat, and RELIEF can retain biological variance even in cases where there is little variance in the covariate of interest (here: participants are approximatively 9 years old, compared toZhang et al., 2023where the age span is broader).

**Table 1.** Performance of machine-learning models to test for covariate variance to be preserved.

	Single sites				Mixed sites			
	Raw	ComBat	CovBat	RELIEF	Raw	ComBat	CovBat	RELIEF
FA								
Age	0.14 (0.06)	0.14 (0.05)	0.14 (0.05)	0.14 (0.06)	0.12 (0.06)	0.13 (0.06)	0.13 (0.06)	0.13 (0.06)
Sex	0.59 (0.02)	0.59 (0.02)	0.59 (0.02)	0.59 (0.02)	0.57 (0.03)	0.58 (0.03)	0.58 (0.03)	0.58 (0.03)
MD								
Age	0.07 (0.05)	0.07 (0.05)	0.06 (0.05)	0.07 (0.05)	0.05 (0.07)	0.05 (0.07)	0.05 (0.07)	0.05 (0.07)
Sex	0.60 (0.04)	0.59 (0.04)	0.59 (0.04)	0.60 (0.04)	0.59 (0.03)	0.59 (0.03)	0.59 (0.03)	0.60 (0.03)

Note: Cells indicate mean and (standard deviation) values over either 8 single sites (single site columns) or over 500 subsamples (mixed site columns). For age values refer to the correlation between true and predicted values, while for sex values represent balanced accuracy scores.

While these first analyses generally support the utility of RELIEF, the results were based on a simulated and homogeneous subsample with limited number of scan sites and manufacturers, which deviates from how the ABCD study sample is used in practice. To test RELIEF’s harmonization capability in a naturalistic scenario, we finally performed harmonization in the full ABCD sample, including imaging data of N = 11,099 subjects across all 29 scan sites (Supplementary Table S1bfor demographics). Next, we deployed the site classification task described above on the same subset of individuals used in the controlled setting ofFigure 1, allowing for direct comparison between a controlled versus naturalistic setting. For the full ABCD sample, we observed that RELIEF showed inferior harmonization performance and substantially more variance in AUC scores compared to ComBat and CovBat (First subplot inFigure 2for FA;Supplementary Fig. S1for MD). To determine what might cause this pattern, we performed a stepwise exclusion of the smallest scan site and repeated the harmonization and classification task respectively. We observed that RELIEF’s harmonization performance increases (i.e., classification AUC decreases) while cumulatively excluding small scan sites, approaching its initial superior performance from the controlled setting (i.e.,Fig. 1) after removing the smallest study sites from the harmonization. While it is generally more difficult to estimate the site effect from small sites, neither ComBat nor CovBat suffered from this performance loss. Similar, even more pronounced patterns were observed for MD (Supplementary Fig. S1).

**Fig. 2.**
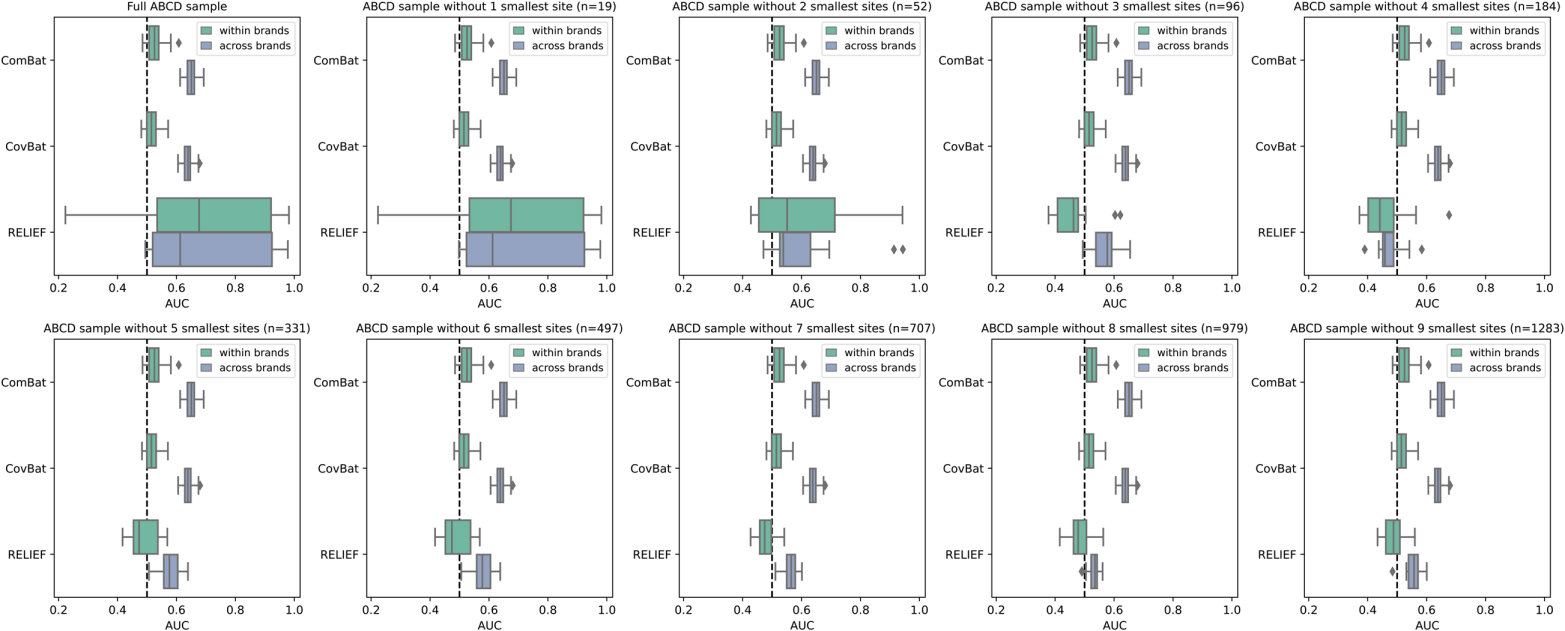
Scan site classification performance across harmonized fractional anisotropy data as a function of the exclusion of scan sites in the ABCD sample. Each subplot depicts a model comparison across harmonization techniques ranging from 0 to 9 excluded sites. Sites were sorted in ascending order and excluded started from the smallest. The full sample (row 1, column 1) included N = 11,099 subjects. N in subplot titles refer to the cumulative sum of subjects excluded per iteration (seeSupplementary Table S2for additional information on the excluded sites). Boxes depict median AUC (vertical line) and interquartile ranges. Note that performance closer to AUC = .5 (chance level) describes a more efficient scan site harmonization and is thus desirable. AUC: area under the curve.

To assess if this concerning harmonization performance drop is due to small study sites in general, or due to the combination of small and large study sites, we performed another analysis in our initial, controlled setting with equally sized subsamples. From the initial N = 500 subjects, we iteratively subsampled for N ∈ {50,100,200,300,400}, and performed harmonization per iteration, followed by the QDA site classification task. We calculated the mean AUC for all within or across brand comparisons, and, as a measure of variance, we additionally calculated the interquartile range (IQR) for within and across brand comparisons. The ideal harmonization would yield consistently low performance, which would be reflected by low mean AUC and small IQR values across trials. As illustrated inSupplementary Figure S2, we observed that most of the RELIEF harmonized data lies close to this “ideal” point, indicating that it performed well in a controlled setting with balanced small samples. Therefore, the alarming performance drop of RELIEF in the full ABCD sample appears to be attributable to the highly unbalanced combination of large and small sites.

Taken together, while the results from our controlled setting with equally sized, large subsamples supported the utility of the RELIEF method, the effects we observed across the full spectrum of ABCD sites warrant caution and call for further development for diminishing unwanted influence of small samples. Since the ABCD study is widely used in the neuroimaging community and many researchers will be interested in finding the best practices for this specific sample, we decided to share our evaluation and provide our code onhttps://github.com/dominikkraft/ABCD_relief, which would allow scholars to re-create our evaluation with specific settings we did not test here. This could be interesting for example when investigating the impact of different covariates to be preserved or removed on overall harmonization performance or when investigating harmonization performance in longitudinal data from this ongoing data collection effort. For the latter, a longitudinal adaption of RELIEF similar to the one developed byBeer et al. (2020)for ComBat might be necessary. Of note, none of the deployed harmonization tools alleviated the across brand site confounds, and therefore there is no one-size-fits-all model bringing relief to the ABCD data at this point. We suggest scholars to evaluate on a study-by-study basis which technique might be the most suitable for their question and sample at hand.

## Supplementary Material

Supplementary Material

## Data Availability

Data used in this study were accessed under data use agreements of the ABCD study cohort. Raw data must not be shared directly by the study authors, but researchers can get access through own data use agreement and use our shared scripts to reproduce the results. All code used in this manuscript is available onhttps://github.com/dominikkraft/ABCD_relief.
