# Supplementary material for: Removing scanner effects with a multivariate latent approach: A RELIEF for the ABCD imaging data?
Source: Imaging Neurosci (Camb). 2024 May 2;2:imag-2-00157. doi: 10.1162/imag_a_00157 (PMC12247595; doi:10.1162/imag_a_00157)
Supplement: Supplementary Material [file imag_a_00157-supp.pdf]

Removing scanner effects with a multivariate latent approach - a RELIEF for the ABCD imaging data?

Dominik Kraft[1*], Gloria Matte Bon[1, 2], Édith Breton[3], Philipp Seidel[1, 4], Tobias Kaufmann[1, 3, 4]

[1] Department of Psychiatry and Psychotherapy, Tübingen Center for Mental Health, University of Tübingen, Tübingen, Germany

[2] Department of Women's and Children's Health, Science for Life Laboratory, Uppsala University, Uppsala, Sweden

[3] Centre for Precision Psychiatry, Division of Mental Health and Addiction, Institute of Clinical Medicine, University of Oslo, Oslo, Norway

[4] German Center for Mental Health (DZPG), partner site Tübingen, Tübingen, Germany

Address for correspondence:

Dominik Kraft, Dr.
Dominik.Kraft@med.uni-tuebingen.de

Department of Psychiatry and Psychotherapy
Tübingen Center for Mental Health
University of Tübingen
Tübingen, Germany

Comment to:

Zhang, R., Oliver, L. D., Voineskos, A. N., & Park, J. Y. (2023). RELIEF: A structured multivariate approach for removal of latent inter-scanner effects. *Imaging Neuroscience (Cambridge, Mass.)*, *1*, 1–16. https://doi.org/10.1162/imag_a_00011

Supplementary Information:

Supplementary Figures:

Figure S1: Scan site classification performance across harmonized mean diffusivity data as a function of the exclusion of scan sites in the ABCD sample.

Figure S2: Influence of sample size on classification performance across different (un)-harmonized data in a controlled subsample of the ABCD with 500 subjects per site.

Supplementary Tables:

Table S1a: Demographics of the subsample used in the first, controlled analysis setting.

Table S1b: Demographics of the full ABCD sample.

Table S2: Site and scanner information for stepwise excluded sites in naturalistic analysis setting.

Supplementary Figures

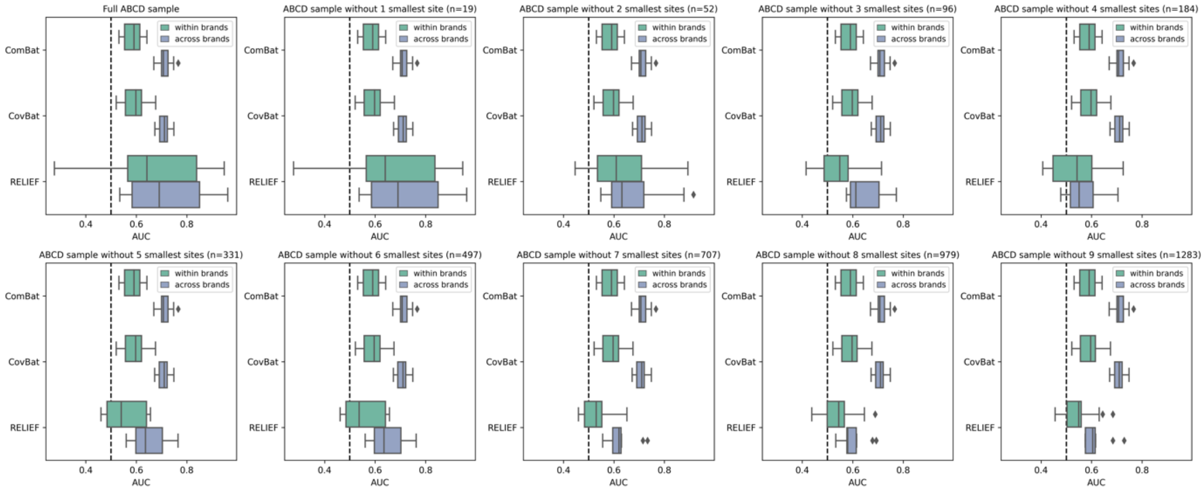

Fig S1. Scan site classification performance across harmonized mean diffusivity data as a function of the exclusion of scan sites in the ABCD sample. Each subplot depicts a model comparison across harmonization techniques ranging from 0 to 9 excluded sites. Sites were sorted in ascending order and excluded started from the smallest. The full sample (row 1, column 1) included N=11099 subjects. N in subplot titles refer to the cumulative sum of subjects excluded per iteration (see Supplementary Table S1 for additional information on the excluded sites). Boxes depict median AUC (vertical line) and interquartile ranges. Note that performance closer to AUC = .5 (chance level) describes a more efficient scan site harmonization and is thus desirable. AUC: area under the curve.

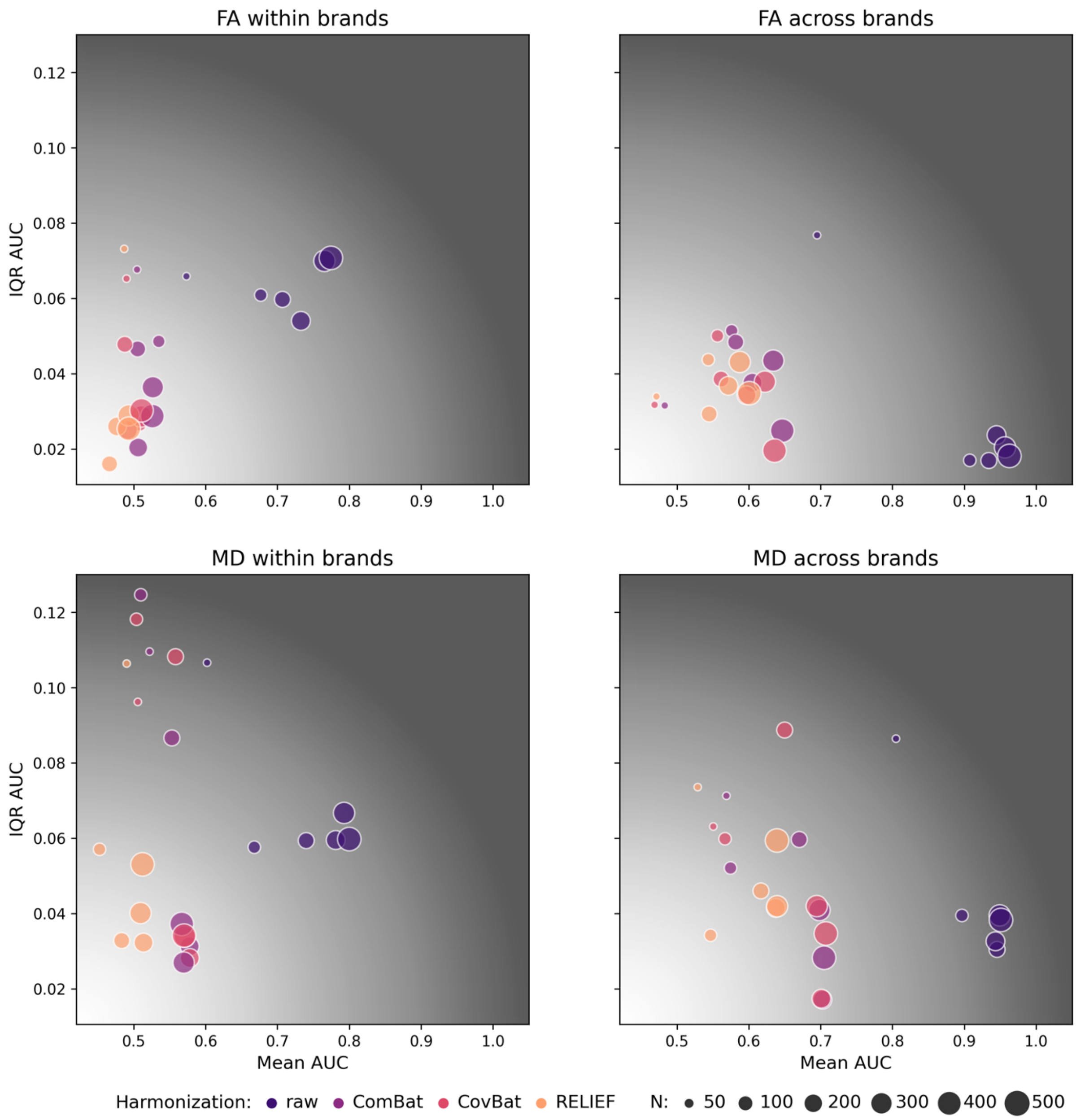

Fig S2. Influence of sample size on classification performance across different (un)-harmonized data in a controlled subsample of the ABCD with 500 subjects per site. Color coding refers to harmonization technique, while scatter size encodes the sample size. The background gradient shows an optimal area (i.e., bright area close to x = 0.5 and a low value on the y-axis), which indicates a close to chance classification performance with little variance across comparisons. Smaller sample sizes tend to introduce higher variance as compared to larger sample sizes, yet this applies across raw and harmonized data. Scatter size refers to subsample drawn from the initial 500 subjects per site. IQR: interquartile range, AUC: area under the curve, Fa: Fractional Anisotropy, MD: Mean Diffusivity.

Supplementary Tables

Table S1a: Demographics of the subsample used in the first, controlled analysis setting.

site	sex		age	
	female	male	mean	SD
HASH1314a204	221	279	120.93	7.61
HASH3935c89e	221	279	118.44	7.90
HASH5b0cf1bb	237	263	118.45	7.60
HASH96a0c182	247	253	119.25	7.15
HASHb640a1b8	225	275	118.88	7.58
HASHc3bf3d9c	250	250	117.77	7.17
HASHd7cb4c6d	236	264	118.58	7.65
HASHe4f6957a	239	261	118.37	7.55

Note: SD= standard deviation. Sex is defined as biological sex assigned at birth. Site refers to the ABCD variable “mri_info_deviceserialnumber”. Age is depicted in month.

Table S1b: Demographics of the full ABCD sample.

site	sex		age	
	female	male	mean	SD
HASHe76e6d72	10	9	118.68	6.99
HASH48f7cbc3	20	13	122.58	6.75
HASH31ce566d	21	23	119.16	6.98
HASH7f91147d	40	48	120.5	7.91
HASHe3ce02d3	74	73	117.15	7.33
HASH69f406fa	80	86	116.69	7.71
HASHc9398971	105	105	121.86	5.66
HASHfeb7e81a	117	155	117.72	7.70
HASH5b2fcf80	147	157	119.45	7.45
HASH65b39280	152	175	118.45	7.60
HASHa3e45734	163	181	119.03	7.57
HASH311170b9	185	181	118.52	7.47
HASH6b4422a7	188	196	118.66	7.58
HASH7911780b	189	211	118.86	7.32
HASH4d1ed7b1	207	208	119.39	7.38
HASH03db707f	208	221	117.74	7.70
HASH4b0b8b05	223	213	117.75	7.86
HASHd422be27	222	228	120.28	6.00
HASH5ac2b20b	255	235	120.90	6.58
HASH11ad4ed5	230	263	122.69	6.60
HASHc3bf3d9c	251	250	117.76	7.17
HASHe4f6957a	254	274	118.43	7.51
HASHd7cb4c6d	255	275	118.58	7.63
HASH1314a204	244	288	120.92	7.57
HASHdb2589d4	260	285	117.73	7.64
HASHb640a1b8	249	299	118.91	7.56
HASH96a0c182	279	274	119.27	7.20
HASH5b0cf1bb	268	293	118.33	7.50
HASH3935c89e	440	544	118.62	7.97

Note: SD= standard deviation. Sex is defined as biological sex assigned at birth. Site refers to the ABCD variable “mri_info_deviceserialnumber”. Age is depicted in month.

Table S2: Site and scanner information for stepwise excluded sites in naturalistic analysis setting.

site	manufacturer	device	subjects excluded
HASHe76e6d72	Siemens	Prisma	19
HASH48f7cbc3	General Electric	Discovery MR750	33
HASH31ce566d	Siemens	Prisma Fit	44
HASH7f91147d	Siemens	Prisma Fit	88
HASHe3ce02d3	General Electric	Discovery MR750	147
HASH69f406fa	General Electric	Discovery MR750	166
HASHc9398971	Siemens	Prisma Fit	210
HASHfeb7e81a	General Electric	Discovery MR750	272
HASH5b2fcf80	General Electric	Discovery MR750	304

Note: The respective sites were excluded row by row, yielding step by step a dataset that contained fewer small sites.
